# Immune Biomarkers at Birth Predict Lower Respiratory Tract Infection Risk in a Large Birth Cohort

**DOI:** 10.3390/pathogens13090765

**Published:** 2024-09-05

**Authors:** Ethan Mondell, Gustavo Nino, Xiumei Hong, Xiaobin Wang, Maria J. Gutierrez

**Affiliations:** 1 School of Medicine, Johns Hopkins University, Baltimore, MD 21205, USA; emondel1@jhmi.edu; 2 Division of Pulmonary and Sleep Medicine, Children’s National Hospital, George Washington University, Washington, DC 20010, USA; gnino@childrensnational.org; 3 Center for Genetic Medicine Research, Children’s Research Institute, Washington, DC 20010, USA; 4 Center on the Early Life Origins of Disease, Department of Population, Family and Reproductive Health, Johns Hopkins Bloomberg School of Public Health, Baltimore, MD 21205, USA; xhong3@jhu.edu (X.H.); xwang82@jhu.edu (X.W.); 5 Division of General Pediatrics and Adolescent Medicine, Department of Pediatrics, Johns Hopkins University School of Medicine, Baltimore, MD 21205, USA; 6 Division of Pediatric Allergy, Immunology and Rheumatology, Department of Pediatrics, Johns Hopkins University School of Medicine, Baltimore, MD 21205, USA

**Keywords:** lower respiratory tract infection, birth cohort, immune biomarkers, cytokines, neonates, cord blood

## Abstract

Lower respiratory tract infections (LRTIs) remain the leading cause of infant morbidity and mortality worldwide and affect long-term respiratory health. Identifying immunological determinants of LRTI susceptibility may help stratify disease risk and identify therapies. This study aimed to identify neonatal immunological factors predicting LRTI risk in infancy. Cord blood plasma from 191 neonates from the Boston Birth Cohort was analyzed for 28 soluble immune factors. LRTI was defined as bronchiolitis, bronchitis, or pneumonia during the first year of life. Welch’s *t*-test demonstrated significantly higher log_10_ transformed concentrations of IL-17 and IFNγ in the LRTI group compared to neonates without LRTI in the first year of life (*p* < 0.05). Risk associations were determined using multivariate survival models. There were 29 infants with LRTIs. High cord blood levels of IFNγ (aHR = 2.35, 95% CI 1.07–5.17), TNF-β (aHR = 2.86, 95% CI 1.27–6.47), MIP-1α (aHR = 2.82, 95% CI 1.22–6.51), and MIP-1β (aHR = 2.34, 95% CI 1.05–5.20) were associated with a higher risk of LRTIs. RANTES was associated with a lower risk (aHR = 0.43, 95% CI 0.19–0.97). Soluble immune factors linked to antiviral immunity (IFNγ) and cytokines mediating inflammatory responses (TNF-β), and cell homing (MIP-1α/b), at birth were associated with an increased risk of LRTIs during infancy.

## 1. Introduction

Lower respiratory tract infections (LRTIs), such as bronchiolitis and pneumonia, are the leading cause of infant morbidity and mortality worldwide [1]. They account for approximately 18% of deaths in children younger than 5 years old, with most deaths occurring during the first 2 years of life [2,3]. Moreover, acute early-life LRTIs are linked to substantial, life-long respiratory burden, including wheezing, asthma, and chronic obstructive pulmonary disease (COPD) [4].

Several immunological markers in cord blood, including IL-1β, IL-2, IL-4, IL-5, IL-6, IL-8, IL-10, IL-13, and IFN-γ, have been linked to LRTI in infants [5,6,7]. However, previous studies often included heterogenous populations and immunological states, and we still lack robust immunological biomarkers for LRTI risk estimation [5,6,7]. Furthermore, our understanding of the interaction between those markers and clinical factors influencing LRTI responses remains incomplete.

In this study, we sought to use a large birth cohort to understand the relationship between baseline levels of 28 soluble immune factors and LRTI during infancy. Specifically, we investigated associations with soluble immune factors that have been implicated in LRTI immune responses in early childhood [5,6,7]. In addition, we examined cytokines involved in host defense to respiratory infections but not yet associated with early-life LRTIs. For example, sentinel myeloid cells in the lungs release TNF-α and IL-1 to upregulate inflammatory responses against pneumococcal pneumonia infection [8,9]. Additionally, IL-17 signaling instructs lung epithelial cells to secrete chemokines necessary for neutrophilic defense against klebsiella pneumonia [10]. IL-12 upregulates IFN-γ signaling to aid in neutrophil defense against pneumonia [11]. TREM-1 positively amplifies TNF-α, and IL-1 and is a strong predictor of pneumonia when found in bronchoalveolar-lavage fluid [12,13].

Accordingly, we analyzed soluble immune factors in the cord blood of neonates along with their clinical characteristics, hypothesizing these would be associated with a different likelihood of early-life LRTIs. Using adjusted Cox regression models, we identified immune markers associated with an increased risk of contracting LRTIs within the first year of life.

## 2. Methods

### 2.1. Study Population

This study was conducted among 191 mother–infant dyads enrolled in the Boston Birth Cohort (BBC) between 2003 and 2006 and examined the relationship between soluble immune biomarkers in cord blood and LRTIs during infancy (0–1 years of age). The BBC is a large, predominantly inner-city birth cohort with over 8600 mother–infant pairs enrolled to date. A detailed description of the parent cohort, as well as methods for selecting participants in the BBC cord blood immune biomarker sub-study, are described in previous work by our group [14,15]. For this study, 927 newborns with available cord blood immune profiling data (28 cytokines and soluble immune factors) were screened for eligibility, and those with complete demographic and available follow-up data during the observation period (0–12 months) were included. A total of 191 infants were included in the final analyses. The Institutional Review Boards at Boston University Medical Center and Johns Hopkins Bloomberg School of Public Health approved the study protocol.

### 2.2. Biomarker Assays

Sample collection was previously described [15]. In brief, umbilical cord blood samples were collected at birth in tubes with EDTA and immediately stored at 4 °C. Samples underwent refrigerated centrifugation at 2500 rpm for 10 min, after which the supernatant was carefully collected to ensure it was free of platelets. Subsequently, each plasma sample was divided into three aliquots and cryopreserved at −80 °C. We assessed 28 immune biomarkers using Flowmetric Luminex xMAP immunoassays (Luminex Corp, Austin, TX, USA). Biomarker quantification was performed using a sandwich immunoassay, employing biotinylated antibodies and phycoerythrin-labeled streptavidin. Further details for biomarker quantification, as well as the limits of detection for each cytokine, were previously described [16]. Additional details on the specific biomarkers are provided in Supplementary Table S1.

### 2.3. Definitions of Outcomes and Covariables

The main outcome of this study was LRTI during infancy, defined as the presence of bronchiolitis, bronchitis, or pneumonia in infants aged 0–12 months. LRTI was identified by ICD-9 or ICD-10 diagnoses recorded in the BBC database. Maternal characteristics analyzed as covariates included mode of delivery (C-section or vaginal), age at delivery, self-reported race and ethnicity, education level, parity (number of previous pregnancies resulting in live births), and pre-pregnancy BMI categorized as either non-overweight (<25 kg/m^2^) or overweight (≥25 kg/m^2^). We also included maternal health conditions, such as smoking during pregnancy, diabetes (none, gestational, or diabetes mellitus), and hypertensive disorders of pregnancy (preeclampsia, eclampsia, and chronic hypertension). Relevant infant variables included gestational age (prematurity), low birth weight (under 2500 g), sex, and breastfeeding status.

### 2.4. Statistical Analyses

During exploratory analysis, we assessed variable distributions, identified missing data, skewness, and outliers, and subsequently removed subjects with incomplete data. For inflammatory markers, we imputed measurements below the level of detection using the lowest level of detection divided by the square root of two. Log10-transformed values were used to mitigate skewness and reduce the impact of outliers. Continuous variables were summarized using means, standard deviations, medians, and interquartile ranges. Demographic and clinical differences between neonates with and without LRTIs were assessed using Chi-square, Student’s *t*-test, and Wilcoxon rank-sum tests. For cord blood immune biomarkers, we employed Welch’s *t*-test to compare levels between infants with and without LRTIs during their first year of life.

Survival models were used to determine risk associations between cord blood biomarkers. The cumulative risk of LRTIs during infancy was estimated for neonates with high (above the 50th percentile) or low (at or below the 50th percentile) immune biomarker concentrations using univariate and multivariate Cox proportional hazards models. See Supplementary Table S2 for median immune biomarker concentrations. Covariables in the adjusted model were limited to sex and prematurity status due to insufficient power to test additional covariables. Time to incident LRTI was defined as the age at which the first LRTI was recorded. Children who did not develop an LRTI were censored at 12 months of age. Survival models were performed using STATA version 14. All other analyses were performed using R version 4.2.3.

## 3. Results

### 3.1. Clinical Characteristics of Mother–Neonate Dyads in the BBC

Our analysis included 191 births occurring between 2003 and 2006 and followed for one year. A total of 29 newborns experienced LRTIs within the first year of life. Among the affected infants, the median age at onset was 5 months (IQR: 3–6). Table 1 presents an overview of the characteristics of the two study groups. Consistent with prior literature, the LRTI group exhibited a higher likelihood of being exclusively bottle-fed (41.4% vs. 20.4%, *p* = 0.012) and born to mothers who smoked during pregnancy (24.1% vs. 5.6%, *p* = 0.001) [17,18,19]. Notably, birth weight, gestational age, race, and parity were not significantly different between the LRTI and no LRTI groups. Similarly, there were no significant differences observed in other infant characteristics (mode of delivery and birth season/year) or maternal factors (race, age at delivery, educational level, BMI, diabetes, eclampsia, and chronic hypertension).

### 3.2. Differentially Abundant Cord Blood Immune Biomarkers between Infants with and without Early-Life LRTI

**Figure 1:**
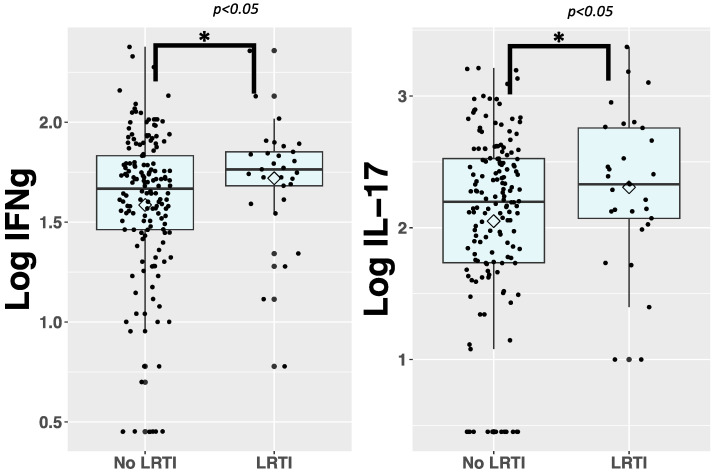
Box and whisker plots presenting immune biomarker concentrations that are significantly different between LRTI and no LRTI groups. We collected 28 immune biomarkers from cord blood of neonates and quantified them using immunoassays. We then log_10_ transformed the data and used Welch’s *t*-test to compare levels of immune biomarkers in the cord blood of neonates with (*n* = 29) and without (*n* = 162) LRTI before one year of life. Biomarker concentrations that are significantly different between comparison groups are presented here. * = *p* ≤ 0.05. The elements of the box and whisker plots represent the median, IQR, and the whiskers are 1.5 times the IQR. Lozenges represent the mean.

We first explored the association between immune biomarkers in cord blood and LRTI susceptibility during infancy using Welch’s *t*-test. To facilitate comparison, cord blood immune biomarker concentrations were log10-transformed. Among the 28 immune biomarkers analyzed, IFNγ and IL-17 exhibited significantly higher concentrations in the LRTI group (Figure 1). The remaining immune biomarkers were not significantly different between groups. When analyzed in categories, there was a larger proportion of newborns with high cord blood levels of IFNγ, TNF-β, MIP-1α, and MIP-1β (defined as cord blood levels in the two higher quartiles, see Supplementary Table S2 for medians) compared with the no LRTI group.

### 3.3. IFN-γ, TNF-β, MIP-1α, and MIP-1β are Associated with a Higher LRTI Risk during Infancy

We next applied multivariate models to examine the relationship between the markers in our study, relevant covariates, and LRTI risk. Specifically, Cox proportional hazards models were employed, adjusting for sex and prematurity as covariates. We conducted univariate and multivariate analyses of the 28 markers included in this study. Infants in the top 50% of IFN-γ levels exhibited a 2.3-fold increase in LRTI risk compared to those in the bottom 50% (adjusted hazard ratio [aHR] = 2.35, 95% confidence interval [CI] 1.07–5.17) (Table 2). Similarly, TNF-β (aHR = 2.86, 95% CI 1.27–6.47), MIP-1α (aHR = 2.82, 95% CI 1.22–6.51), and MIP-1β concentration (aHR = 2.34, 95% CI 1.05–5.20) conferred significantly higher LRTI risk, while RANTES had the inverse relationship (aHR = 0.43, 95% CI 0.19–0.97). In contrast, the levels of other immune factors did not exhibit statistical significance beyond the predefined threshold.

## 4. Discussion

The major findings of our study are that increased IFNγ, TNF-β, MIP-1α, and MIP-1β levels at birth are associated with a higher risk of LRTI during infancy while RANTES is associated with a lower risk. Although previous studies have identified associations between cord-blood cytokines and LRTI in early childhood, ours investigates a wide array of immune biomarkers at baseline in an inner city, predominantly minority birth cohort, and identifies predictors of early-life LRTI risk in this population [5,6,7].

In our study, clinical factors were associated with the occurrence of future LRTIs [18,20]. The links between breastfeeding status and maternal smoking and LRTI are consistent with previous research [21]. Of note, certain variables previously implicated in early-life LRTI risk, such as parity, gestational age, and maternal weight, did not exhibit significant disparities in our analysis [19,22]. Considering that prior research in larger subsets of the BBC has identified parity, gestational age, and maternal weight as associated with early-life LRTI [23], it is possible that our current analysis is underpowered to detect differences in those variables. Additionally, regarding prematurity effects, the majority of preterm infants in our analysis (46 out of 57) were born mildly premature (between 33 and 36 weeks of gestational age). This might obscure the higher risk of lower respiratory tract infections (LRTIs) and respiratory issues commonly observed in infants born at or before 32 weeks of gestation (severely or extremely preterm infants) who often bear the brunt of respiratory diseases [24].

Our cohort and the scale of our immune biomarker panel allowed for the identification of multiple cytokines associated with LRTI. We noted elevated levels of IFN-γ, TNF-β, MIP-1α, and MIP-1β in neonates who experienced LRTIs during the first year of life. These biomarkers showed an association with an increased risk of LRTI during infancy in further analyses using adjusted survival analyses. Cox regression demonstrated neonates with high baseline IFN-γ, TNF-β, MIP-1α, and MIP-1β levels had greater than a two-fold higher risk of contracting LRTI within the first year of life, independently of gestational age and sex, which are well-known clinical risk factors for early-life LRTIs. Due to study power limitations and variable amounts of missing data, we omitted covariables such as maternal smoking, childcare, or breastfeeding.

IFN-γ is secreted by T cells and is involved in Th1 responses [25,26]. IFN-γ recruits macrophages and monocytes and plays a central role in antiviral immunity [27,28]; infants with high IFN-γ levels during RSV infection have a milder course than infants with low levels [29]. However, high IFN-γ in mice has also been shown to attenuate antibody response during RSV infection [30]. Based on previous literature, we expected IFN-γ levels to be lower in the LRTI group [6,7]. Intriguingly, our study reveals the opposite. One possible explanation is that prior studies evaluated cytokines collected from mononuclear cells stimulated with phytohemagglutinin or other immunogenic antigens, thus collecting cytokines in a stimulated state, while our study evaluated circulating cytokines at birth, absent of stimulation. As these previous investigations were functional studies, our observational study cannot be compared. It may be the case that the total change in cytokine concentration is more immunologically important in combating pathogens than is the cytokine concentrations at any one state. Thus, babies who naturally have higher levels of these circulating cytokines when not stimulated may be less able to mount a robust immune response, as immune cells may become desensitized over time. Indeed, T cell exhaustion is well documented, and overproduction of pro-inflammatory cytokines in a non-infectious state could lead to downregulation of cytokine receptors and a diminished immune response during infection. [31,32,33,34,35,36]. Excessive interferon signaling may also be associated with delayed development of adaptive, virus-specific T-cell and antibody responses [37]. Our results demonstrate that there may be a subset of infants with high cord blood IFN-γ concentrations that are more predisposed to LRTI during infancy.

High cord blood levels of macrophage inflammatory proteins 1α (MIP-1α) and 1b (MIP-1β), also known as CCL3 and CCL4, were associated with LRTIs during infancy in our study. These findings are consistent with previous observations in adults where elevated serum levels of these chemokines during respiratory viral infections, such as COVID-19, influenza, and Middle East respiratory syndrome (MERS), have been linked to increased disease severity and the presence of acute respiratory distress syndrome (ARDS) and thus, proposed as biomarkers of adverse outcomes [38,39]. However, unlike previous studies where elevated levels of MIP-1α and MIP-1β were identified as markers of severe disease during the course of a clinical illness, our discovery of higher levels of these chemokines at birth, associated with future risk, is novel. MIP-1α and MIP-1β are chemokines secreted by various immune cells, including activated lymphocytes and macrophages, contributing to the recruitment of inflammatory cells in the lungs and airways during respiratory infections, possibly exacerbating inflammation and tissue damage. We speculate that in newborns, elevated levels of these cytokines may indicate early activation of inflammatory pathways due to prenatal or perinatal environmental stimuli, which may influence later respiratory outcomes [20,40]. Further characterizing the association between MIP-1α, MIP-1β, and LRTI risk could help guide preemptive strategies to identify and mitigate risks associated with early-life LRTIs.

Transforming growth factor-beta (TGF-β) also emerged as a significant predictor associated with an increased risk of LRTIs in our study. TGF-β is crucial in regulating immune responses to respiratory infections, serving as a modulator of both innate and adaptive immunity [41]. It attenuates the antiviral IFN-β response, thereby facilitating viral replication in influenza infections [41]. In the context of respiratory syncytial virus (RSV), rhinovirus, and parainfluenza, TGF-β also impairs the innate antiviral immune response. For example, TGF-β produced by epithelial cells facilitates RSV replication by inducing cell cycle arrest. However, during the later stages of infection, TGF-β assumes a protective role by regulating adaptive immunity and promoting mucosal IgA responses [41]. In our study, elevated baseline levels of TGF-β may be linked to a more subdued antiviral response to respiratory viruses, which are the most prevalent pathogens in early life. However, further research is warranted to elucidate the mechanisms underlying this association.

Our study also showed a higher baseline level of RANTES is associated with lower early-life LRTI risk. RANTES is most closely involved with Th1 response and is pro-inflammatory [42]. It is secreted by T cells (among other cells) and is chemotactic for eosinophils, monocytes, macrophages, and other T cells [43]. Although to our knowledge, ours is the first study to show this association in human newborns at birth, previous murine models have shown RANTES knockout to cause poor cytokine production, higher inhibitory receptors associated with T cell exhaustion, and elevated levels of viral load in the setting of LCMV infection [44]. Moreover, the interaction of RANTES with its cognate receptor CCR5 is necessary for viral clearance during influenza infection through activation of resident macrophages, NK and T cell recruitment, and the establishment of immunological memory in the respiratory tract [45,46,47,48,49]. Aligned with these findings, our study suggests that baseline levels of RANTES may be important in mounting an immune response against viral respiratory infections, which are the most prevalent in infancy. Nonetheless, additional research is necessary to establish a definitive causal link in human infants.

Our study has limitations that warrant acknowledgment. Primarily, this is a large study to examine immunological profiles at birth as predictors of LRTI risk in infancy. However, our ability to evaluate the influence of immunological and environmental factors shaping immunity after birth was limited. We also were limited in accounting for certain potential confounding clinical variables due to a limited sample size. Furthermore, analyzing a non-randomized observational cohort inherently carries a risk of unmeasured confounding variables. Our study only includes physician-diagnosed cases, which may enrich our study population with neonates with the most severe cases of LRTI in infants who required medical evaluation, limiting the generalizability of our results outside of that group. However, the most severe cases are also most likely to result in adverse neonatal outcomes, making this a group of special interest. Additionally, the use of ICD9/ICD10 codes to ascertain patient outcomes may introduce inaccuracies to the data that increase the risk of bias. Our study population is also primarily an inner-city, racial minority birth cohort, so there could be some limitations in generalizing our study’s findings. However, our birth cohort provides valuable insights for populations historically neglected in research. Lastly, our dataset lacked information around the causative pathogen in LRTIs, which is an important factor when studying immunological profiles in association with LRTIs, but previous work reveals that viral respiratory infections are the most common cause of pneumonia and bronchiolitis in children under 5 years of age [50]. For instance, RSV accounted for 24% of LRTI hospitalizations in children younger than 5 years of age between 1997 and 2006 [51,52].

Nonetheless, our study advances the broader understanding of neonatal LRTI susceptibility. Moreover, the identification of potential predictive immune biomarkers holds promise for refining risk stratification methodologies. This work underscores the imperative for continued research in validation studies and the exploration of novel variables to better understand the multifactorial contributions to early-life LRTIs.

## Figures and Tables

**Table 1:** Clinical Characteristics and Demographics of Mother-Child Dyads. Summary of clinical and demographic characteristics by LRTI and no LRTI status during the first year of life. There were 162 infants without an LRTI in the first year of life and 29 with an LRTI in the first year of life. There was a higher proportion of babies that were bottle-fed in the LRTI group. No other infant characteristics were significantly different between groups. A significantly greater proportion of infants in the LRTI group were born to mothers who smoked continually during pregnancy. No other maternal characteristics were significantly different between groups. Notably, there were significant missing data on childcare attendance. IQR = Interquartile Range, SD = standard deviation, AA = African American, AAPI = Asian American and Pacific Islander, HS = High School, BMI = Body Mass Index, GDM = Gestational Diabetes, DM = Diabetes Mellitus. Bolded *p*-values indicate statistical significance (*p* < 0.05).

	No LRTI before 12 Months (*n* = 162)	LRTI before 12 Months (*n* = 29)	*p*
**Age of First LRTI, median, (IQR), months**	-	5 (3–6)	-
**Sex**			
Female, *n* (%)	78 (48.1)	12 (41.4)	0.5013
Male, *n* (%)	84 (51.9)	17 (58.6)	
**Mode of delivery**			
C-section, *n* (%)	45 (27.8)	8 (27.6)	0.9831
Vaginal, *n* (%)	117 (72.2)	21 (72.4)	
N/A, *n* (%)	-	-	
**Gestational age**			
≥37 weeks, *n* (%)	115 (71)	19 (65.5)	0.5532
<37 weeks, *n* (%)	47 (29)	10 (34.5)	
**Birth weight mean, ± SD, grams**	2985.92 ± 648.5	2905.7 ± 743.6	0.589
**Breastfeeding status**			
Ever breastfed, *n* (%)	125 (77.2)	16 (55.2)	**0.01235**
Never breastfed, *n* (%)	33 (20.4)	12 (41.4)	
N/A, *n* (%)	4 (2.5)	1 (3.4)	
**Birth season**			
Winter, *n* (%)	45 (27.8)	8 (27.6)	0.965
Spring, *n* (%)	28 (17.3)	4 (13.8)	
Summer, *n* (%)	45 (27.8)	9 (31)	
Fall, (%)	44 (27.2)	8 (27.6)	
**Birth year**			
2003, *n* (%)	86 (53.1)	21 (72.4)	0.1616
2004, *n* (%)	75 (46.3)	8 (27.6)	
2005, *n* (%)	1 (0.6)	0 (0)	
**Race**			
White, *n* (%)	6 (3.7)	4 (13.8)	0.1924
AA & Haitian, *n* (%)	108 (66.7)	17 (58.6)	
Hispanic, *n* (%)	31 (19.1)	5 (17.2)	
AAPI and Other, *n* (%)	17 (10.5)	3 (10.3)	
**Age at delivery mean, ± SD, years**	28.9 ± 6.5	27.9 ± 7.3	0.5011
**Education level**			
Less than some secondary school, *n* (%)	53 (32.7)	11 (38.0)	0.814
HS diploma, *n* (%)	53 (32.7)	10 (34.5)	
some college or more, *n* (%)	55 (32.7)	8 (27.6)	
N/A, *n* (%)	3 (1.9)	-	
**Parity**			
No children, *n* (%)	79 (48.8)	17 (58.6)	0.3283
Children, *n* (%)	83 (51.2)	12 (41.4)	
**BMI median, (IQR), kg/m²**	25.3 (21.9–28.6)	26.8 (22.3–29.7)	0.4021
Non-overweight (BMI < 25), *n* (%)	73 (45.1)	11 (37.9)	0.3777
Overweight (≥25), *n* (%)	78 (48.1)	17 (58.6)	
N/A, *n* (%)	11 (6.8)	1 (3.4)	
**Smoking during pregnancy**			
No, *n* (%)	148 (91.4)	22 (75.9)	**0.001164**
Yes, *n* (%)	9 (5.6)	7 (24.1)	
N/A, *n* (%)	5 (3.1)	-	
**Diabetes**			
No, *n* (%)	146 (90.1)	26 (89.7)	0.874
GDM, *n* (%)	9 (5.6)	2 (6.9)	
DM, *n* (%)	7 (4.3)	1 (3.4)	
N/A, *n* (%)	-	-	
**Eclampsia**			
No, *n* (%)	144 (88.9)	25 (86.2)	0.5177
Yes, *n* (%)	16 (9.9)	4 (13.8)	
N/A, *n* (%)	2 (1.2)	-	
**Chronic hypertension**			
No, *n* (%)	144 (88.9)	25 (86.2)	0.7513
Yes, *n* (%)	18 (11.1)	4 (13.8)	
N/A, *n* (%)	-	-	
**Median age at start of childcare attendance, (IQR), years [*n*]**	0.96 (0.25–1.96), [72]	0.75 (0.33–2.0), [22]	0.4307

**Table 2:** Adjusted Immune Biomarker Predictors of LRTI. Adjusted Cox proportional hazards models predict LRTI occurrence by immune biomarker concentration. Cox proportional hazards analysis, adjusted by preterm status and sex, were performed for all immune biomarkers. Newborns with high concentrations of IFNγ, MIP-1α, MIP-1β, and TNF-β at birth demonstrated a significantly elevated risk of contracting LRTI in the first year of life compared to newborns with low concentrations. Newborns with high concentrations of RANTES at birth demonstrated lower risk of LRTI during infancy. IL-17, as well as the remaining immune biomarkers investigated in this study, demonstrated no associations with the risk of contracting an early-life LRTI. High cytokine levels: >50th percentile, low cytokine levels: ≤50th percentile (Supplementary Table S2). CI = Confidence Interval. Bolded *p*-values indicate statistical significance (*p* < 0.05).

Immune Biomarker	Hazard Ratio	95% CI	*p*
**High [IFN-γ]**	2.35	1.07–5.17	**0.03**
**High [IL-17]**	1.21	0.57–2.54	0.621
**High [MIP-1α]**	2.82	1.22–6.51	**0.025**
**High [MIP-1β]**	2.34	1.05–5.20	**0.037**
**High [RANTES]**	0.43	0.19–0.97	**0.042**
**High [TNF-β]**	2.86	1.27–6.47	**0.011**

## Data Availability

The data presented in this study are available from Xiaobin Wang (xwang82@jhu.edu), Principal Investigator of the Boston Birth Cohort, upon reasonable request and after review and approval of the Institutional Review Board.

## Supplementary Materials

The following supporting information can be downloaded at: https://www.mdpi.com/article/10.3390/pathogens13090765/s1, Table S1: Table Showcasing the Immune Biomarkers Investigated in this Study. Table S2: Immune Biomarker Concentration Medians of Cytokines Found to be Significant in the Study.
