# Supplementary material for: Immune Biomarkers at Birth Predict Lower Respiratory Tract Infection Risk in a Large Birth Cohort
Source: Pathogens. 2024 Sep 5;13(9):765. doi: 10.3390/pathogens13090765 (PMC11435078; doi:10.3390/pathogens13090765)
Supplement: Supplementary file 1 [file pathogens-13-00765-s001.zip › Supplementary Table 1.pdf]

Supplementary Table 1: Table Showcasing the Immune Biomarkers Investigated in this Study. 28 Immune biomarkers were studied, using immunoassay with antibodies sourced from 7 companies.

Immune Biomarker	Antibody Source	Recombinant Antigen Source
brain-derived neurotrophic factor (BDNF)	R&D Systems	R&D Systems
C-reactive protein (CRP)	DAKO	DAKO
granulocyte-macrophage colony-stimulating factor (GM-CSF)	R&D Sytems	R&D Systems
interferon-γ (IFN-γ)	BioSource	BioSource
Interleukin-1β (IL-1β)	R&D Systems	R&D Systems
IL-2	BD Biosciences Pharmingen	BD Biosciences Pharmingen
IL-4	BD Biosciences Pharmingen	BD Biosciences Pharmingen
IL-5	R&D Systems	R&D Systems
IL-6	BD Biosciences Pharmingen	BD Biosciences Pharmingen
IL-8	R&D Systems	R&D Systems
IL-10	BD Biosciences Pharmingen	BD Biosciences Pharmingen
IL-12	BD Biosciences Pharmingen	BD Biosciences Pharmingen
IL-17	R&D Systems	R&D Systems
IL-18	MBL	MBL
monocyte chemoattractant protein 1 (MCP-1)	BD Biosciences Pharmingen	BD Biosciences Pharmingen
macrophage migration inhibitory factor (MIF)	R&D Systems	R&D Systems
macrophage inflammatory protein 1α (MIP-1α)	Biotrend/R&D Systems	Biotrend
macrophage inflammatory protein 1β (MIP-1β)	R&D Systems	R&D Systems
matrix metalloproteinase 9 (MMP-9)	R&D Systems	R&D Systems
neurotrophin 3 (NT-3)	R&D Systems	R&D Systems
neurotrophin 4 (NT-4)	R&D Systems	R&D Systems
regulated on activation, normal T-cell expressed and secreted (RANTES, or CCL5)	R&D Systems	R&D Systems
soluble IL-6 receptor α (sIL-6rα)	BD Biosciences Pharmingen	peprotech
soluble tumor necrosis factor receptor I (sTNF-RI)	R&D Systems	R&D Systems
transforming growth factor β (TGF-β);	R&D Systems	R&D Systems
tumor necrosis factor α (TNF-α)	R&D Systems	R&D Systems
tumor necrosis factor β (TNF- β)	R&D Systems	R&D Systems
Triggering receptor expressed on myeloid cells 1 (TREM-1).	R&D Systems	R&D Systems
