# Supplementary material for: Immune Biomarkers at Birth Predict Lower Respiratory Tract Infection Risk in a Large Birth Cohort
Source: Pathogens. 2024 Sep 5;13(9):765. doi: 10.3390/pathogens13090765 (PMC11435078; doi:10.3390/pathogens13090765)
Supplement: Supplementary file 1 [file pathogens-13-00765-s001.zip › Supplementary Table 2.pdf]

Supplementary Table 2: Immune Biomarker Median Concentrations by Group

	**No LRTI Before 12 months (n=162)**	**LRTI Before 12 Months (n=29)**	**All Babies (n=191)**	***P***
**IL-17, median, (IQR), ng/L**	157.5 (280.5)	214 (454)	163 (290)	0.1233
**TNF-β, median, (IQR), ng/mL**	152.5 (181.5)	217 (218)	161 (192)	**0.05166**
**IFN-γ, median, (IQR), ng/L**	46.5 (39)	58 (23)	50 (40)	0.05784
**MIP-1α, median, (IQR), ng/L**	446 (430.25)	556 (313)	477 (445.5)	0.2238
**MIP-1β, median, (IQR), ng/L**	692 (611.5)	810 (410)	719 (604)	0.4329
**RANTES, median, (IQR), ng/mL**	43.35 (29.6)	37.1 (30.7)	43.5 (30)	0.4735
